# Reasons for Journal Impact Factor Changes: Influence of Changing Source Items

**DOI:** 10.1371/journal.pone.0154199

**Published:** 2016-04-22

**Authors:** Tobias Kiesslich, Silke B. Weineck, Dorothea Koelblinger

**Affiliations:** 1 Laboratory for Tumour Biology and Experimental Therapies (TREAT), Institute for Physiology and Pathophysiology, Paracelsus Medical University, Strubergasse 21, 5020, Salzburg, Austria; 2 Department of Internal Medicine I, Paracelsus Medical University / Salzburger Landeskliniken, Müllner Hauptstrasse 48, 5020, Salzburg, Austria; 3 Research Office, Paracelsus Medical University, Strubergasse 21, 5020, Salzburg, Austria; Universidad de Las Palmas de Gran Canaria, SPAIN

## Abstract

Both the concept and the application of the impact factor (IF) have been subject to widespread critique, including concerns over its potential manipulation. This study provides a systematic analysis of significant journal Impact Factor changes, based on the relative contribution of either one or both variables of the IF equation (i.e. citations / articles as the numerator / denominator of the quotient). A cohort of JCR-listed journals which faced the most dramatic absolute IF changes between 2013 and 2014 (ΔIF ≥ 3.0, n = 49) was analyzed for the causes resulting in IF changes that theses journals have experienced in the last five years. Along with the variation by number of articles and citations, this analysis includes the relative change of both variables compared to each other and offers a classification of `valid`and `invalid`scenarios of IF variation in terms of the intended goal of the IF to measure journal quality. The sample cohort features a considerable incidence of IF increases (18%) which are qualified as `invalid`according to this classification because the IF increase is merely based on a favorably changing number of articles (denominator). The results of this analysis point out the potentially delusive effect of IF increases gained through effective shrinkage of publication output. Therefore, careful consideration of the details of the IF equation and possible implementation of control mechanisms versus the volatile factor of number of articles may help to improve the expressiveness of this metric.

## Introduction

Despite the well-known concerns and critique of the meaning and validity of the (Journal) Impact Factor ((J)IF) [1, 2], it continues to be a widespread instrument for the assessment of scientific output. Originally developed as a guideline for librarians to compare journal quality within particular scientific subject categories [3, 4], the IF is being applied likewise to measure and compare the scientific output of individuals or institutions. Consequently and despite attempts to develop more significant metrics (e.g. [5]), the IF continues to play a dominant role in academic career development [6].

A journal´s IF in a given year results from the equation of citations to this journal within the particular year to articles published in this journal during the two previous years, divided by the number of citable articles (“substantive articles and reviews” [4]), the so-called source items, which were published in these two precedent years.

Concerns on the validity of the IF or its actual meaning (for overviews, see e.g. [7, 8]) include the effects of ‘influencing variables’ (e.g. article types and type of discipline [9], language bias [10], citation misconduct [11], IF inflation [12]), and conceptual limitations (e.g. unequal distribution of citations [13], ‘not dividing like with like’ [8, 14, 15] and the Matthews effect [8, 16]). Additionally, frequent practices to influence the IF have been observed and denominated by some authors as the “impact factor game” [2] or “top-ten JIF manipulation” [17]–some of them have also been listed, with different grades of ethicality, as recommendations to “new editors” to improve the IF of their journal [18]. These different forms of IF alterations–alleged or putative manipulations–predominantly attempt to boost citations, either by means of direct editorial influence on reference lists within publications [17, 19], or by applying tactical measures which allow to expect an increase of citations: nine out of the top-ten IF manipulations identified by Falagas et al. explicitly aim at increasing the numerator of the IF equation. The remaining one targets at the non-citability of published articles in order to decrease the denominator [17].

As the number of citations expressed in the numerator and the number of articles counted in the denominator equally influence the IF, the question arises whether actually observable IF changes are only or mainly attributable to a change of citations–as it should be. The aim of this work is to provide a systematic analysis of significant journal IF changes, based on either one or both variables of the IF equation. Based on a cohort of JCR-listed journals which faced the most dramatic IF changes from 2013 to 2014 (absolute ΔIF ≥ 3.0, n = 49 journals), we investigated the causes responsible for these IF changes into further detail. Since the mere observation of the IF over time by itself provides no information on these causes, the current analysis necessarily includes the relative change of both variables compared to each other in addition to the variation by number of articles and citations.

Based on this assumption, we classified the observed IF changes as ‘valid’ or ‘invalid’ increases and decreases of the IF relating to the explanatory power as a measure of (changing) quality or ‘impact’ of a given journal.

## Materials and Methods

### Data collection and inclusion criteria

**Fig 1:**
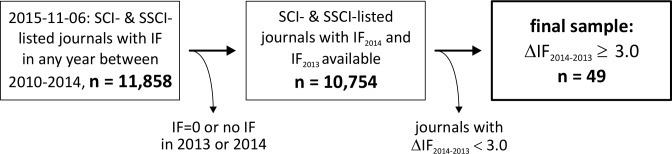
Sample selection. From all journals listed with an Impact Factor (IF) in the Science Citation Index (SCI) or Social Sciences Citation Index (SSCI), those were selected for further analysis which showed a change of the IF (ΔIF) equal or greater than a threshold of 3.0.

In November 2015, a list of all journals (n = 11,858) was derived from the annual journal citation report (JCR) published by Thomson Reuters via the software Toad for Oracle Base® 11.5 (Oracle Corp., Redwood Shores, CA, USA), containing both the Science and the Social Science edition and including all journals that have had an IF at least in one of the years since 2010. Journals featuring an IF of 0.000 or no IF at all in one of the years 2013 and 2014 were excluded. From 10,754 journals remaining, those journals with an absolute IF increase or decrease of 3.0 or more between 2013 and 2014 were selected for further analysis (n = 49)–the procedure for sample selection is depicted in Fig 1.

As a basis for subsequent in-depth analysis, the relevant data determining the IF for the years 2010–2014, i.e. numbers of articles and citations tracing back until 2008 were manually extracted from the journal citation report web-database (Thomson Reuters).

Contrary to the before-mentioned exclusion of journals featuring no IF or an IF of 0.000 in 2013 or 2014, we did not exclude journals which lack an IF within one or more of the earlier years of analysis (2010, 2011 or 2012). These are typically either new journals or such which underwent renaming. In these cases, we started calculating IF deltas from two years on after being listed first in the JCR in order to exclude artificial IF deltas owing to new listings or renaming only.

### Data handling and analysis

The journals included as well as all analyses were performed in an anonymized fashion since explicitly naming them is of no relevance for the intention and conclusion of the current study. After sorting the 49 journals for their ΔIF (2014–2013), an abstract but unique identifier (1–49) was assigned to each journal. All calculations were performed in Microsoft® Office Excel® and data visualizations were generated using OriginPro 9.1 (OriginLab Corp., Northampton, MA, USA) and Corel Designer® X5 (Corel Corp., Ottawa, Canada).

## Results

### Overall IF deltas and sample cohort

**Fig 2:**
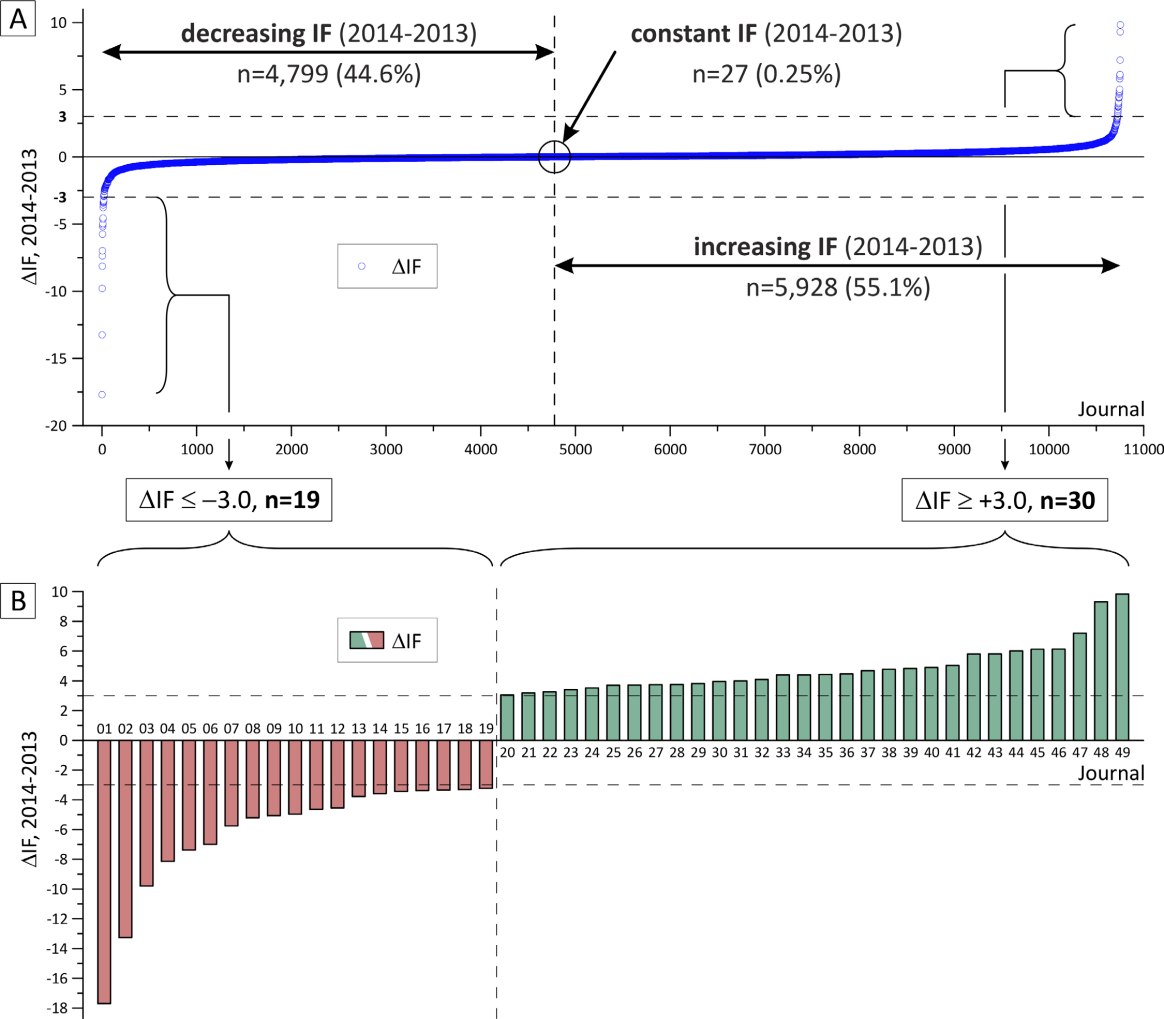
Range of Impact Factor changes (ΔIF) for 2014 versus 2013. (A) ΔIFs for all 10,754 journals with a listed IF for 2013 and 2014. (B) ΔIFs for the journals (n = 49) in the selected study cohort (threshold ΔIF ≥ 3.0). In- and decreasing IFs are highlighted green or red, respectively. The 49 journals in the sample cohort are subsequently identified by a unique identifier (#1 - #49).

As summarized in Fig 2, our sample cohort included journals with an absolute ΔIF ≥ 3.0. Among all JCR-listed journals (n = 11,858; November 2015), the majority (n = 5,928; 55.1%) feature an IF increase between 2013 and 2014, while the IF of 4,799 journals (44.6%) decreased and 27 journals remained unchanged (0.25%; Fig 2A). In our cohort (n = 49), the number of journals with increasing IF is higher than that with decreasing IF: 30 journals show a positive ΔIF (61.2%) and 19 (38.7%) an IF decrease (negative ΔIF, Fig 2B). As illustrated in Fig 2B, positive ΔIF values range between +3.05 (journal #20) and +9.83 (journal #49) while decreasing IF between years 2013 and 2014 range between -3.23 (journal #19) and -17.70 (journal #1).

### Classification of IF changes

**Fig 3:**
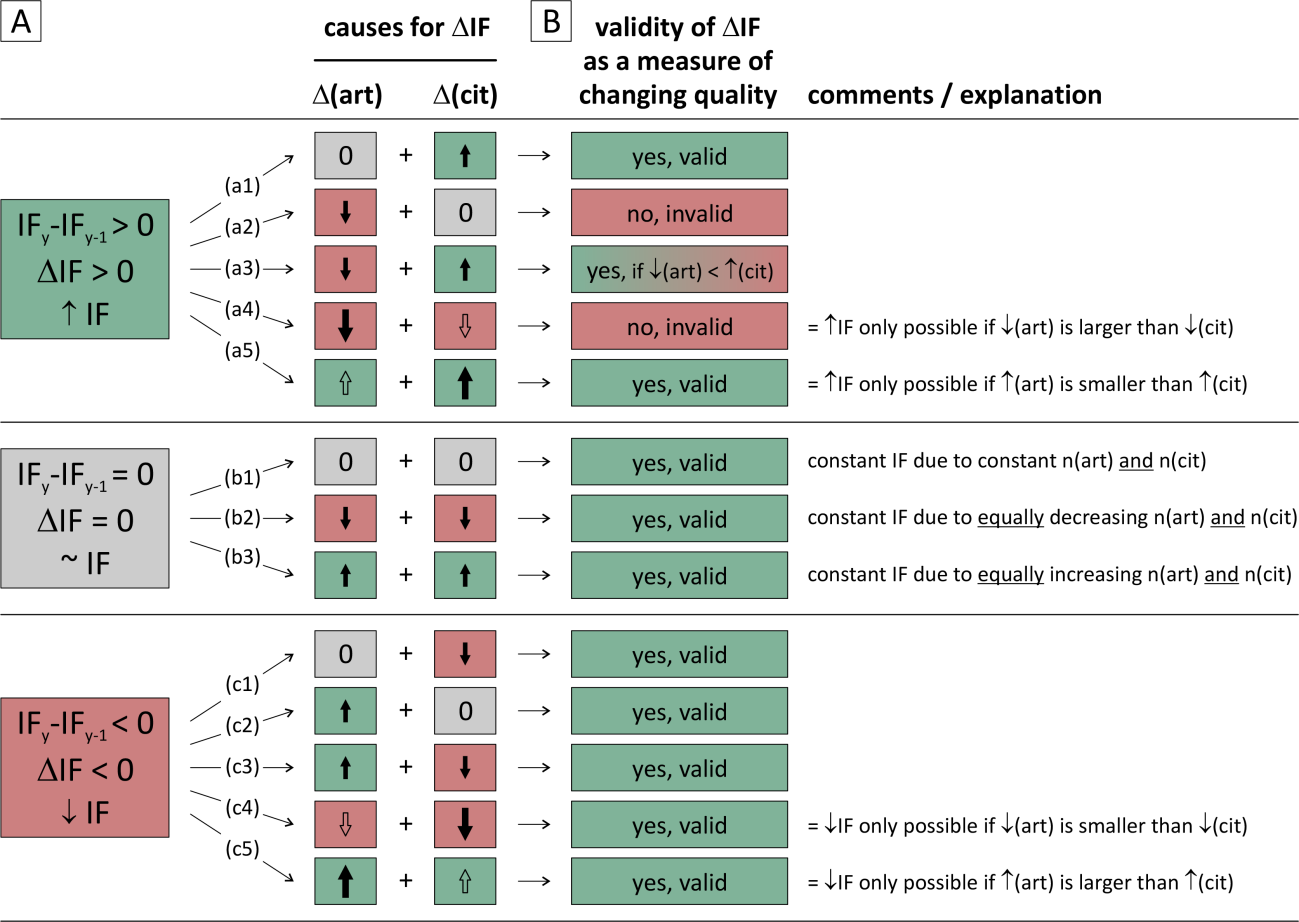
Possible scenarios explaining a changing Impact Factor (IF). (A) Based on theoretical considerations regarding the potential influence of the variables (citations and article numbers) on the final calculation (i.e. the IF quotient), 13 scenarios are possible which either cause an increased (a1-a5), constant (b1-b3) or decreased IF (c1-c3). In those scenarios where necessary, the size of the arrows indicate the relative importance of the changes (articles (Δ(art)) versus citations (Δ(cit))). (B) The validity of these IF changes as a parameter of (changing) journal quality is categorized and referred to as either ‘valid’ (highlighted green) or ‘invalid’ (highlighted red). See text for further explanation.

Garfield´s proposition that the average citation rate per article is a surrogate measure of a journal´s quality [4] only holds true provided the citation count (indicating the reception of a publication (its “impact”); the numerator) is directly proportional to the calculated metric (IF). However, since the number of articles (citable source items, the denominator) may change too, via theoretical considerations we first identified 13 possible scenarios of how either the number of articles or citations influence the IF. As shown in Fig 3, each five scenarios are possible accounting for an increasing and decreasing IF while three scenarios yield a constant IF (referred to as scenarios a1-a5, c1-c5 and b1-b3 in Fig 3A, respectively).

From our proposed classification (weighting) of scenarios, IF changes in both directions involving increasing citations are mostly qualified as ‘valid’ in terms of their significance as a parameter of journal quality. IF changes involving increasing number of articles are valid in all scenarios (a5, b3, c2, c3, c5). Likewise, a decrease by number of articles which leads to decreasing IF (c4) is plausibly inherent to the concept of the IF as are scenarios resulting to a constant IF (no change at all (b1), equally decreasing (b2) or increasing (b3) articles and citations). However, scenarios with IF increases which are merely based on decreasing numbers of published articles (a2) or relatively stronger decrease by articles than by citations (a4) are being qualified as ‘invalid’ since such IF increases are based only on shrinkage of the journal’s output and only reflect favorable changes in the IF equation.

Out of the ten variations resulting in an IF change shown in Fig 3, five have the same impact on the IF regardless of the relative amount of variations of each variable concerned: a constant numerator or denominator with the other variable changing invariably results in an IF change, regardless of the amount of change of that variable (a1, a2, c1, c2). Likewise, a reduction in citation numbers accompanied by a growing number of articles always results in an IF decrease (c3). We qualify all these variations as valid except the scenario of an increasing IF mentioned above which occurred merely due to a shrinkage of the number of published articles (a2).

The remaining five scenarios are dependent on the change of both variables of the IF equation *relative to each other*: a simultaneous increase or decrease of both the number of articles and citations can lead to IF variations in either direction, depending on which variable change is relatively more profound than the other. Therefore, the simultaneous increase of both article and citation numbers constitutes two different, but equally valid scenarios resulting either in an IF increase or decrease (a5, c5). Similarly, a cumulative decrease of both constituents can lead to either an increase or decrease of the IF (a4, c4). However, while the IF decrease (c4) is a valid scenario in this case, we qualify an IF increase (a4) as clearly invalid, as outlined above.

A decreasing number of articles combined with a growth by number of citations (a3) always results in a positive ΔIF. In the subsequent analysis, we consider this scenario as ‘valid’ provided the increase of citations exceeds the decrease by number of articles, otherwise it constitutes an ‘invalid’ IF increase.

### Validity of IF changes in the sample cohort

During the period of our analysis, the 49 journals are characterized by relative citation changes ranging between -92.9% and +3,135.0% between one year and the subsequent one, as shown in Table 1. Relative changes of article numbers range from -84.6% to +583.3% and relative ΔIF occurred within -90.0% up to +1,621.9%. Therefore, in the current journal cohort, not only changes in citations but also such in numbers of published articles (source items) vary considerably throughout subsequent years–together resulting in partly dramatic changes of the journals’ IF.

**Table 1:** Observed maximum changes of citations, articles and Impact Factors.

		maximum decrease (%)^a^	maximum increase (%)^a^
2014–2013	Δ(Citations)	-92.9	3135.0
	Δ(Articles)	-60.0	583.3
	Δ(IF)	-86.7	1621.9
2013–2012	Δ(Citations)	-90.0	322.5
	Δ(Articles)	-66.7	169.0
	Δ(IF)	-90.0	206.3
2012–2011	Δ(Citations)	-42.9	177.5
	Δ(Articles)	-66.7	128.8
	Δ(IF)	-41.4	274.9
2011–2010	Δ(Citations)	-88.9	105.5
	Δ(Articles)	-84.6	96.4
	Δ(IF)	-68.2	186.4

^a^…relative to preceding year

**Fig 4:**
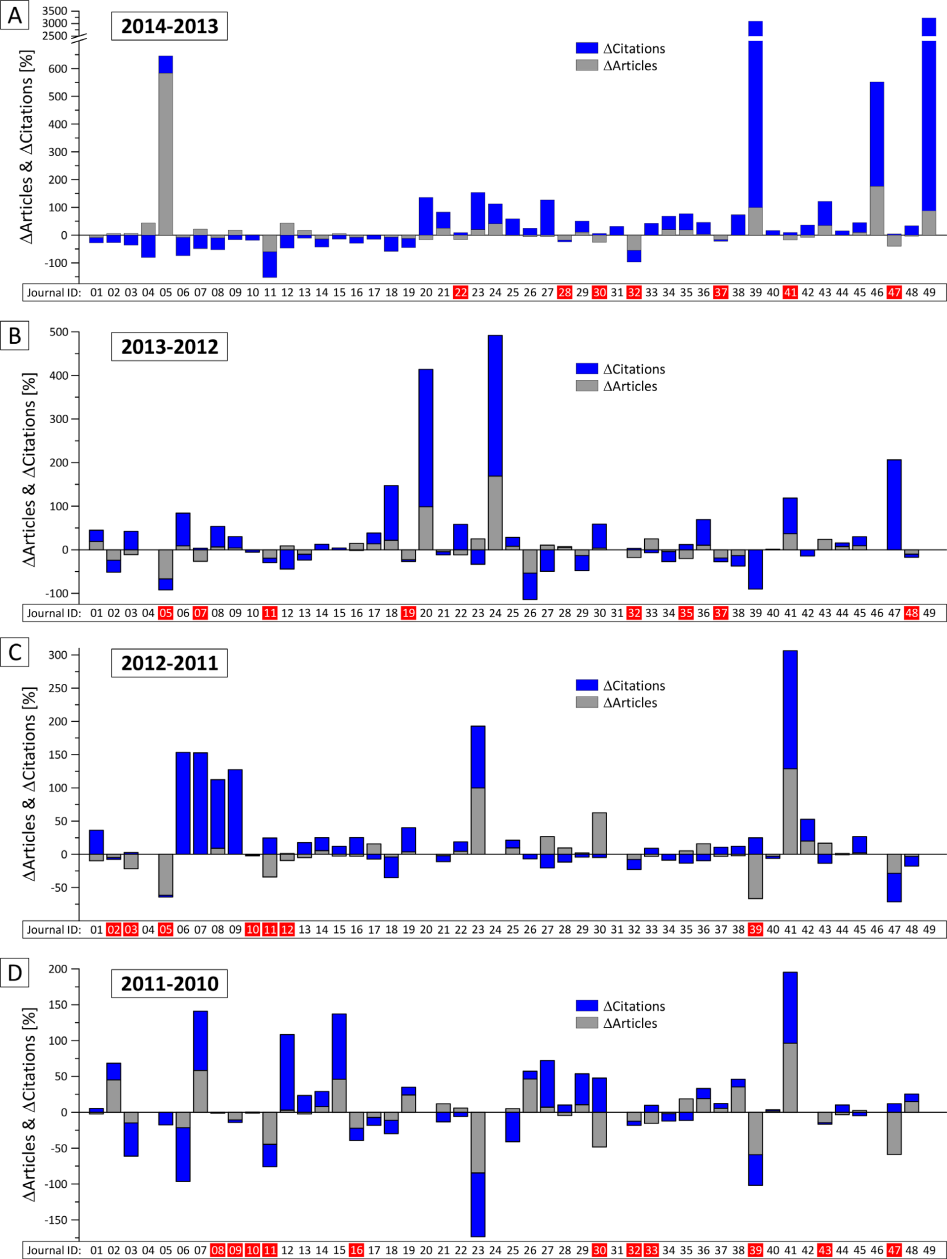
Annual changes of the Impact Factor (IF) and its variables (citation versus article numbers). For the annual changes (A-D) of the IF, the relation of relative changes of the number of citations versus articles is shown in each diagram by blue and grey columns, respectively. Each column shows the relative change (%) in relation the numbers of citations or articles in the preceding year. Below each diagram, the ΔIF for each annual step is classified ‘invalid’ as defined in Fig 3 and indicated by a red box behind the journal ID. The 49 journals in the sample cohort are identified by a unique identifier (#1 - #49).

The scheme obtained from our systematic classification approach (Fig 3) applied on the selected sample of journals (n = 49 with ΔIF ≥ 3.0 between 2013 and 2014, Fig 2C) identifies 14.3% (n = 7) invalid IF increases (Fig 4A). Four of these journals (#22, #30, #41, #47) faced an increase of citations which was relatively smaller than the reduction of published articles (scenario a3) and three journals (#28, #32, #37) experienced a decrease of citations which was smaller than the decrease of articles (scenario a4).

**Fig 5:**
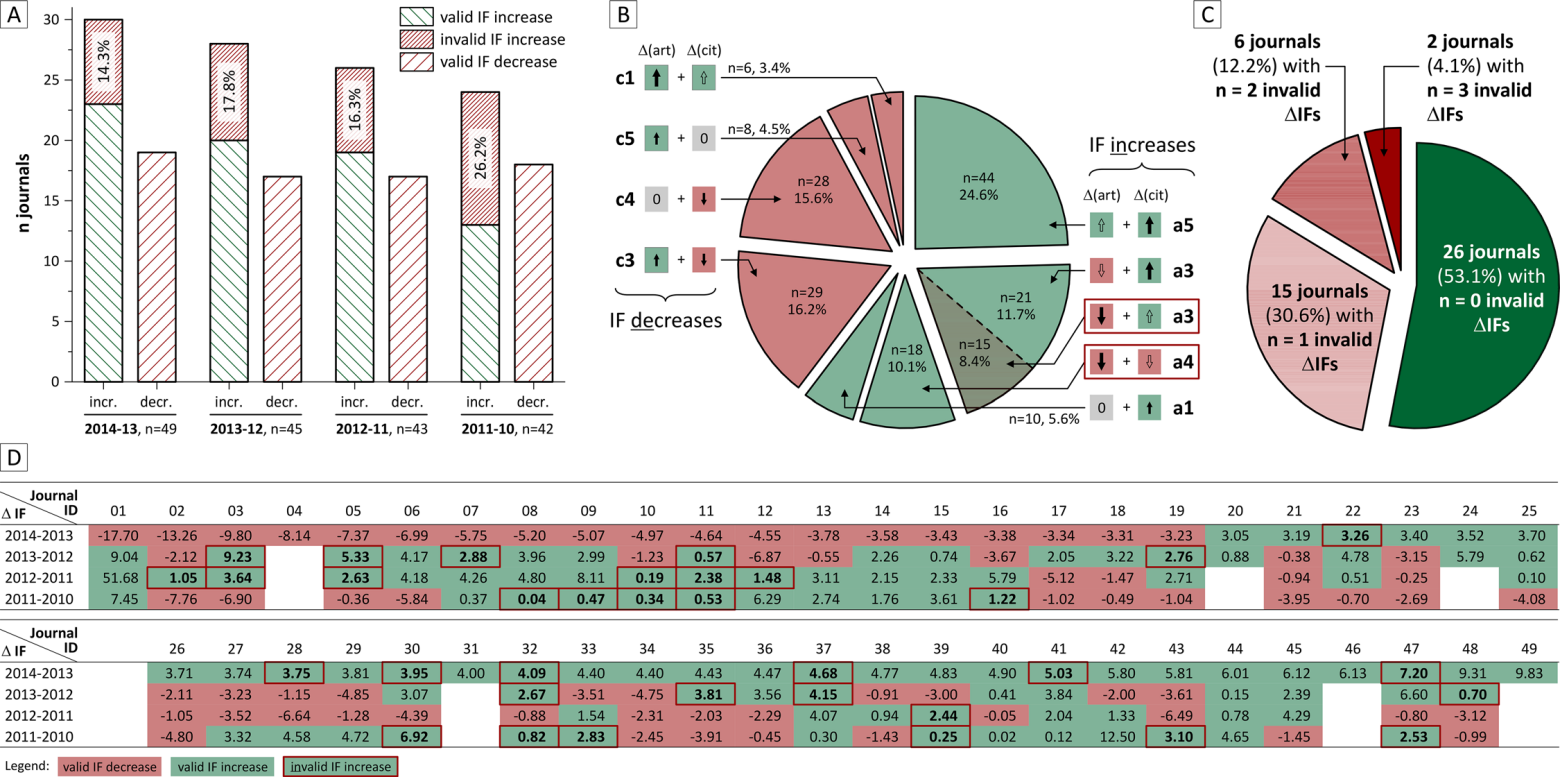
Summary of observed scenarios / variants of changing Impact Factor (IF). (A) IF increases (left bars) and decreases (right bars) for each annual step 2010–11 to 2013–14. IF increases are further classified as valid or invalid according to the classification in Fig 3. (B) The observed IF changes (n = 179) over the four annual steps are classified according to the scenarios explained in Fig 3 and illustrated for their relative frequency. Invalid IF changes (a3 and a4) are highlighted by red boxes. (C) Overall distribution of journals regarding to the validity of their IF changes. (D) Longitudinal development of each journal in the sample cohort. IF increases, IF decreases and invalid IF increases are indicated by green / red color or red boxes, respectively.

As summarized in Fig 5A, our analysis of the same journal cohort for the two precedent annual ΔIFs (Fig 4B and 4C, Fig 5A) shows similar results: between 2012 and 2013, 17.8% (n = 8) of 45 evaluable journals and between 2011 and 2012, 15.6% (n = 7) of 43 evaluable journals feature invalid IF increases. As in 2013–14, the distribution between ‘invalid’ scenarios with both decreasing article and citation numbers (a4) and those facing relatively stronger decrease of article numbers than increase of citations (a3) is about half-half: 5:3 for 2012–13 and 3:4 for 2011–12. Analyzing ΔIFs between 2010 and 2011 identifies 26.2% (n = 11) invalid IF increases. Forty-two journals had been allocated an IF in these years, seven of which experienced decreasing numbers of both articles and citations (a4) and four journals had a relatively smaller increase of citations than decrease of articles (a3).

Fig 5B outlines the distribution of all IF-changing scenarios throughout the entire timespan of our analysis and (n = 179 ΔIFs in total) shows that 10.1% of invalid IF increases were caused by a more pronounced reduction of articles compared to the reduced citation counts. In other words, within our sample of 49 journals, it occurred 18 times within five years that a journal´s IF increased even though the journal shrinked both by numbers of published articles and citations (a4). Together with the 15 cases of invalid IF increases resulting from relatively stronger decrease of article numbers than increase of citations (a3), the number of invalid IF increases sums up to n = 33, i.e. 18.4% invalid ΔIFs of a total of n = 179 evaluable ΔIFs.

The longitudinal dynamics of IF changes (Fig 5D) shows the distribution of invalid IF increases for each journal. Due to the relatively small number of investigated annual IF changes (four), derivation of clear trends for individual journals is probably not justified. Nevertheless, as summarized in Fig 5C, even though more than half of the journals in our sample (n = 26) had no invalid IF increases throughout the timespan of our analysis, 15 journals (30.6%) feature an invalid IF increase once, 6 journals twice and 2 journals showed invalid increases for three times during the analyzed four annual IF changes.

## Discussion

Several aspects of critique versus the concept of the IF as such and its various kinds of uses and misuses have been expressed previously [7–13] (see also introduction section). Furthermore, several authors have discussed potentially problematic effects of the quotient the IF calculation is based on–i.e. the division of total citations (“to virtually any item” [14]; numerator) by the ‘articles’ (citable items; denominator) [8, 14, 15]. To our knowledge, there is currently no other systematic analysis which explores the different scenarios of ‘invalid’ IF increases based on comparing changes in both parts of the quotient, their incidence and distribution over a specific sample or even all JCR-listed journals.

Althouse et al. [20] have investigated the temporal development of the IF for 4,300 journals and found about 80% of journals to have increased their IF between 1994 and 2005. Similarly, our analysis covering 10,754 journals identified a larger proportion of journals featuring an increasing versus decreasing IF between 2013 and 2014 (55.1% versus 44.6%). While confirming the reported trend of inflating IF over time [12], the smaller proportion of increasing journals (compared to [20]) might be due to the shorter period of analysis in our study. Interestingly, Althouse et al. identified the increase in citations in the reference lists of published papers as the most important contributor to the IF inflation.

The sample selection of the present work includes the most extreme cases of changing IFs (absolute ΔIFs ≥ 3.0 between 2013 and 2014). For several reasons, we assume that this inclusion criterion is useful for the current study: first, the analysis in this particular sample will reveal a clearer result and better validity of the distinction between valid and invalid IF increases than calculating increases and decreases in the rather continent range of deltas around 0 which would be selected by a threshold based on relative (%) IF changes. Second, journals featuring larger IF changes are potentially more affected by both gain and loss of reputation, and still there are no control mechanisms, deletions from JCR or any other consequences following repeated invalid IF increases at stake. Finally, third, our analysis additionally includes IF changes during four subsequent years and thus provides an overview not only of the most extreme deltas, but also of previous, potentially smaller changes. This longitudinal analysis ensures that punctual extreme changes are not being overvalued and allows to validly observe the gradual IF development of all 49 journals.

The definition of the scenarios a2, a4 (and a3, if applicable) as ‘invalid’ could be questioned: by its nature as an arithmetic mean value, increases of the IF by a mere reduction of the denominator implicate that a given (probably steady) number of citations distributes over a reduced number of articles–which would thus be a legitimate result meaning ‘less articles acquire relatively more citations’. However, as the distribution of the citations a journal acquires is known to be highly skewed [2, 13, 21–23], such IF increases would require that the reduction of the denominator is not evenly distributed over all papers a journal publishes. In other words, such an IF increase would require that only lowly cited papers are excluded for the denominator while highly cited papers are still published and counted. Relating to its originally intended use as a metric on the journal level [4, 24, 25], we consider such changes as not meaningful and thus ‘invalid’.

While the ongoing discussion predominantly circulates around the issue of citations [4], our analysis clearly points out the potentially problematic issue of IF increases resulting from decreasing numbers of articles. While changing the denominator is discussed as a journal’s option to potentially manipulate the IF [8, 14, 15], the standard interpretation and reception of the IF allocates quality to changes by numbers of citations which leaves alterations by numbers of articles as a blind spot. The fact that over a timespan of four years, a remarkable 15–25% of journals feature invalid IF increases bears evidence of the delusive effect of the IF (and its changes), even when applied on its original concept to measure journal quality [1, 26].

Generally, only one variable of the IF equation is potentially valid to express changes in journal quality, i.e. the number of citations. The number of articles, however, equally affects the result and thus may constitute a distorting as well as threatening factor for the validity of the IF. For individual journals, maximum decreases by articles of 60% and up to 84.6% between one year and the subsequent one could be identified in our analysis. We believe that such extreme decreases are not only to be deducted from editorial measures, i.e. reduction of published articles and reject confirmatory or negative studies to predominantly cover highly citable (‘trendy’) manuscripts [17]. The fact that decisions on the citability (as “substantive, scholarly articles”) of each item are made by Thomson Reuters by considering the bibliographic and bibliometric characteristics [27] adds to this volatility. As pointed out by Krell F.T. [28], several journals have litigated (legitimate) discussions with Thomson Reuters on the appreciation of articles as citable and thus contributing to the denominator–with partly significant effects on the eventually calculated IF [2, 29]. Similarly, if journals re-categorize a portion of published items as ‘citable’ in the denominator, the IF can undergo dramatic (negative) changes: in 1997, the IF of Lancet decreased from about 17 to 12 after dividing the section ‘Letters’ into ‘Correspondence’ and (citable) ‘Research Letters’ [14].

As calculated by Opthof et al. [30], changing the criterion for acceptance–i.e. only accepting manuscripts with a “100% priority score” assigned by the reviewers–could increase the IF by about 40%. In line with our conclusions, the authors note in this context that such a policy (accepting only 100%-scored manuscripts) would reduce the journal’s content below 30% [30]. Regardless of the actual cause of a change in the denominator (changing allocating practice by Thomson Reuters or effective growth or shrinkage of the journal’s output), the current data suggest that the apparent IF increase several journals experienced needs further analysis to be fully understood.

Based on the assumption that the number of articles (denominator), contrary to the number of citations, is primarily subject to the journal’s editorial decisions, the denominator of the IF equation might bear the risk to be exploited as a potential playground for manipulation. Further studies would have to analyze whether ‘intentional reduction of the denominator’ should be included in the list of top-IF-manipulations [17]. While significant alterations of citation numbers led to temporary exclusions of journals from the JCR (e.g. Acta Foliatrica Logopaedica, [31, 32]), we could not find any reference in the editorial information to the JCR, nor any examples of journals which were deleted due to (significant, repetitive or else suspicious) IF increases based on shrinking numbers of articles.

Another result derived from our analysis relates to the frequency and concentration of invalid IF increases: only slightly more than half of our sample journals never showed any invalid IF increases over the course of four years, while n = 8 journals have two or three invalid IF increases. Six journals did not show any valid IF increase, but feature alterations of invalid IF increases and IF decreases throughout the entire timespan of our analysis. Only a further journal-specific and detailed analysis could clarify whether these journals continuously reduced their number of articles, the number of citable articles or whether this change is due to the IF publisher’s article allocation practice. Conclusive statements on the legitimacy of individual journal’s policies regarding the IF equation’s denominator additionally would need to be observed over a longer time period than the current analysis.

Taken together, the frequency of invalid IF increases identified in our cohort is a phenomenon eminently worth considering and requires new control mechanisms by part of Thomson Reuters which currently do not seem to be at stake [31]. If not directly provided by the publisher of the JCR, at least librarians in charge of selecting journals for future subscription might be interested to take a more detailed look into the numerical changes of all factors contributing to the IF calculation. Even so, during the last release of the JCR (InCites^TM^) earlier this year it became more convenient to display the changes in ‘citable items’ (IF denominator) for each journal.

## Conclusions

In conclusion, far-reaching decisions based on journal quality and the IF as its most popular metric, e.g. for continued or new journal subscriptions, have to consider the details of the IF equation which consists not only of numbers of citations but also numbers of published articles. While the emphasis of use and interpretation of the IF lies predominantly on the citation part of the equation, the equally volatile number of articles needs, in our view, more careful consideration.
